# Taxonomy of Anomaly Detection Techniques in Crowd Scenes

**DOI:** 10.3390/s22166080

**Published:** 2022-08-14

**Authors:** Amnah Aldayri, Waleed Albattah

**Affiliations:** Department of Information Technology, College of Computer, Qassim University, Buraydah 52571, Saudi Arabia

**Keywords:** crowd, anomaly detection, abnormal behavior, surveillance system, CCTV

## Abstract

With the widespread use of closed-circuit television (CCTV) surveillance systems in public areas, crowd anomaly detection has become an increasingly critical aspect of the intelligent video surveillance system. It requires workforce and continuous attention to decide on the captured event, which is hard to perform by individuals. The available literature on human action detection includes various approaches to detect abnormal crowd behavior, which is articulated as an outlier detection problem. This paper presents a detailed review of the recent development of anomaly detection methods from the perspectives of computer vision on different available datasets. A new taxonomic organization of existing works in crowd analysis and anomaly detection has been introduced. A summarization of existing reviews and datasets related to anomaly detection has been listed. It covers an overview of different crowd concepts, including mass gathering events analysis and challenges, types of anomalies, and surveillance systems. Additionally, research trends and future work prospects have been analyzed.

## 1. Introduction

The World Health Organization (WHO) clarifies significant gathering events as any occurrence, whether planned or unplanned, that attracts a substantial number of participants to strain the neighborhood, city, or nation hosting the event’s planning and response resources [1]. The heterogeneous composition of the crowd in terms of color, age, language, and culture presents several administrative issues for local organizers focused on ensuring the event’s efficient management. Administrative authorities are more concerned with understanding the crowd mechanics that explain what could harm large crowds [2]. An anomaly detection system is a monitoring program that automatically identifies and considers the signs of abnormal or irregular actions directly [3]. With the widespread usage of video surveillance techniques, manual evaluation of vast quantities of video data gathered from crowd surveillance CCTV cameras has become complicated, time-consuming, and ineffective in the case of large crowds [4]. It requires workforce and continuous attention to decide if the captured actions are normal or abnormal. Therefore, an automatic anomaly detection functionality is necessary for surveillance systems to accurately identify and detect anomalies in crowd scenes [5]. Detecting abnormal behaviors rapidly and automatically in crowded environments is significant for improving safety, preventing risks, and guaranteeing quick response. Anomaly detection in surveillance systems is critical for assuring safety, security, and in some cases, the prevention of possible disasters [6]. Anomaly detection intends to discover the anomalies in a quick time automatically. Recently, intelligent monitoring systems have become crucial for effective crowd management. Due to their importance, computer vision, video analysis, and automated crowd anomaly detection have become popular research topics.

### Contribution

A comprehensive overview of the crowd concept, abnormal human behavior, and surveillance systems have been discussed. A new taxonomic organization of the recent developments in abnormal human behavior detection techniques for large-scale (Danse) crowds has been proposed to identify subfields that are still unexplored or that are seldom approached from the perspective of deep learning. A wide range of recent deep learning approaches to detecting anomalies has been covered. It includes research papers and reviews published in the time interval from 2011 to 2022. Moreover, this review focuses on studying the human crowd, specifically human abnormal behavior.

## 2. Crowd and Mass Gathering Event

**Figure 1:**
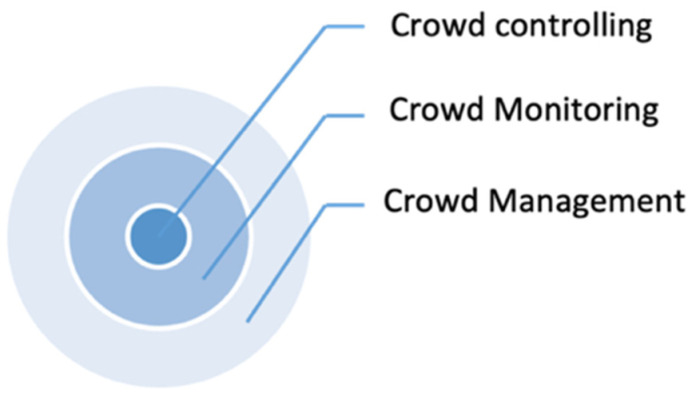
Crowd analysis concepts.

A large-scale event is crowded and attracts people from multiple locations with diverse cultural backgrounds, which generates significant management, control, and communication challenges due to their diversity [7]. These large gatherings are potentially dangerous for the public. Numerous physical characteristics describe crowd behavior, including the direction of motion, velocity, energy, and interaction force [8]. The field of crowd analysis includes three general concepts or levels: crowd management, crowd monitoring, and crowd control, see Figure 1. Crowd management is defined as using techniques to plan and manage mass gathering events before, during, and after the event. It ensures the safety of people, good event planning and managing, predicts and prevents unexpected issues, and prepares initial plans for emergencies. Crowd monitoring provides the opportunity to estimate crowd dynamics, detect and predict possible risks, track, support virtual simulation of crowd behavior, and develop automated systems [9]. Globally, security and event management agencies are beginning to realize the importance of crowd monitoring, considering the growing concern about public safety. Crowd control is a public security practice and actions taken during the situation to prevent abnormal behavior when such as fights, riots, or crowd crushes occur. An automated crowd scene analysis involves counting, tracking, and identifying the behaviors of a large crowd of individuals occupying the same physical space [10]. An estimate of the number of people in a certain area is known as crowd counting.

A critical aspect of crowd safety is crowd action recognition, which recognizes the different actions of an individual or group of individuals. The ability to track objects in crowded video sequences is critical to interpreting visual scenes [11]. In Islam, Hajj is considered one of the five pillars and a duty that all physically able, healthy, and financially capable people must fulfill once in a lifetime. Pilgrims worldwide arrive in one place over five days to perform religious rites. In the Muslim lunar calendar, this begins on day 8 of Zulhijjah and ends on day 13, Zulhijjah [12]. This annual mass gathering event is considered one of the biggest in the world. Since the number of pilgrims attending these events has increased over the years, many challenges could occur, such as overcrowding at the sites resulting in congestion, stampedes, damages, loss of pilgrims, violations, and fatalities [1]. The Kingdom of Saudi Arabia seeks to provide pilgrims with the best possible Hajj experience by providing infrastructure, safety, security mechanisms, and numerous other amenities to manage these crowds. However, it is still seeking more tools for this task [13].

## 3. Crowd Analysis

**Figure 2:**
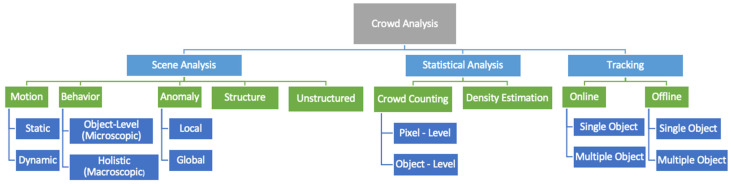
Proposed taxonomy for crowd analysis.

Most research works divide crowd analysis into two major branches: crowd statistics and crowd behavior analysis. This study introduces another branch, the tracking approach. Figure 2 illustrates the newly proposed taxonomy for crowd analysis.

- **Scene Analysis**: Automatic video analysis is called video analytics, and it can detect and analyze temporal and spatial events. The usefulness in finding anomalies in real-time, monitoring crowds, detecting pedestrians, and tracking vehicles make video scene analysis an active research topic. The CCTVs distributed in crowded public areas facilitate the process of analyzing the motion, behavior understanding, anomaly detection, and determining the type of the crowd, whether it is structured or unstructured.
- **Statistical Analysis**: Crowd density estimation and crowd counting are examples of statistical analysis, which involves analyzing patterns and trends in quantitative data. The number of people per meter can be used to calculate crowd density. While crowd counting is a method of counting how many people are present in a space. These estimations are effective in controlling the flow of the crowd in a specific area and avoiding overcrowding, accidents, and stampedes.
- **Tracking**: Object tracking is the process of determining the location of moving objects over time [14]. An object can be tracked online or offline, and one object or several objects can be tracked simultaneously. The changes in features over time can be used to track anomalies detected by object detection.

## 4. Crowd Scene Analysis Challenges

**Figure 3:**
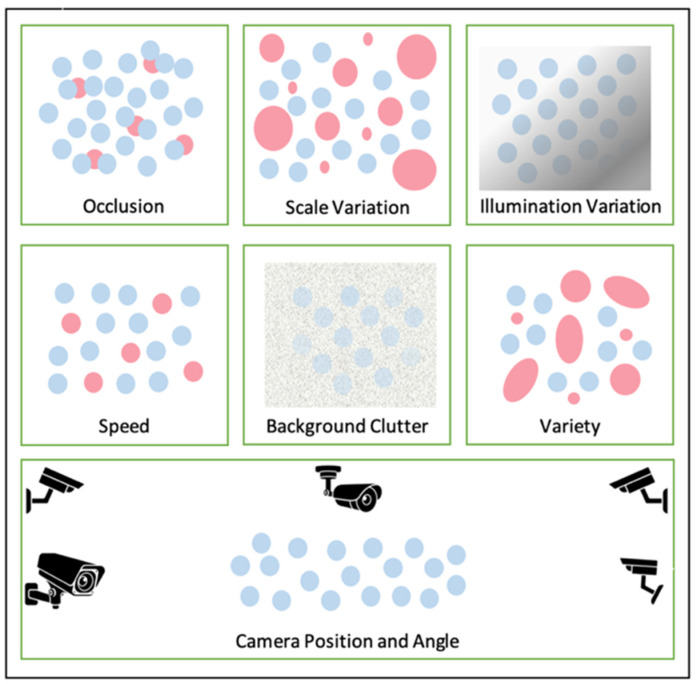
Crowd scene analysis challenges.

Occasionally, some moving entities in videos do not appear clearly to the observer in some circumstances. There are diverse kinds of challenges, as illustrated in Figure 3:

- **Occlusion**: this happens when two or more objects come too close jointly and seem to merge, which leads to the system losing track of the trackable object or tracking the wrong object because of overlapping [15].
- **Scale Variation**: it occurs when there is a wide range of sizes of the tracked objects, which causes the tracking system to lose precise tracking.
- **Illumination Variation**: refers to the variation in the quantity of origin light mirrored on an image and can be caused by changes in lighting, shadows, or noise.
- **Speed**: while objects in a scene often move at different speeds, the predictor should recognize objects in motion videos accurately by being fast during prediction.
- **Background Clutter**: it refers to the existence of large numbers of objects in the image, which makes it difficult for a detector to recognize individual objects due to their non-uniform arrangement. There is a possibility that objects that need identifying will blend into the background, making them difficult to detect.
- **Variety**: occurs when an object has more than one shape and size.
- **Camera Position and Angle**: it is possible to have inconsistencies in perspective due to different angles and camera positions, as well as the tilting and up-and-down motion of the camera.

## 5. The Concept of Anomaly

The word anomaly comes from the Greek word “anomolia”, which indicates irregular or uneven patterns [16]. In the data mining and statistics communities, anomalies are also known as abnormalities, deviants, and outliers [17]. It can be defined as an unusual pattern that does not conform to expected behavior or place. For textual data, the anomaly can be detected by plotting the data; the data points that are greater than or less than other data are referred to as anomalies or outliers, which is inconsistent with other data. On the other hand, for videos or image data, the anomaly can be identified by analyzing and understanding the behavior or patterns of objects in that area; the object that behaves differently from expected patterns is an anomaly object. Anomaly detection refers to the detection and localization of patterns or any behavior that does not correspond to expectations. A person may exhibit abnormal behavior in public alone or as part of a group. Thousands of pilgrims gather simultaneously in the Hajj area, which is an illustration of a heavily populated place. Different abnormal activities could happen, such as congestion, walking against the pedestrian path, standing in places not designated for standing and obstructing the movement of pilgrims, sitting in places other than those designated for sitting, and running and scrambling at the gates and the train station. Moreover, violence is a representation of abnormal behavior, which is a physical force that affects the surrounding area and people; it can be detected through a smart surveillance system that helps to control the safety of the environment and limit violations and other accidents [18].

## 6. Anomaly Detection

Monitoring public security often involves the detection of abnormal behavior in surveillance videos of crowds. Anomaly detection in crowded scenes refers to the detection of irregularities, abnormalities, or discovering patterns that are out of alignment with normal behavior in images or video sequence data. In [19], anomaly detection is described as identifying patterns that are extremely distinct from the rest. According to [20], anomaly detection refers to the identification of crowd movements, where the abnormal behaviors in crowded locations usually emerge as crowd commotion. The detection of anomalies aims to identify and categorize anomalies in given datasets [21]. Anomaly detection can be classified into three categories: supervised, unsupervised, and semi-supervised. The dataset that has both data and labels can be used for supervised anomaly detection. The labels determine the type of event, whether it is “normal” or “abnormal.” Unlabeled datasets are employed for the unsupervised anomaly detection method. The unsupervised method considers that most of the events in the dataset are normal and otherwise assumed an anomaly. In situations where the dataset has not been completely labeled nor unlabeled, meaning that some data are labeled, and some are unlabeled, semi-supervised anomaly detection techniques are used. As a rule, anomaly detection procedures assess the patterns in the available normal data, illustrate them, and then model them in order to uncover new patterns in the new data [22]. Surveillance systems [23,24,25,26,27], intrusion detection [28,29,30], fraud detection [31,32], and health monitoring [33,34,35] are just a few of the domains where anomaly detection has applications.

## 7. Types of Anomalies

Basically, the term anomaly refers to anything that is unusual, irregular, or uncharacteristic and differs from the normal event [36]. An anomaly can be broken down into three types: point anomalies, extended anomalies, and collective anomalies.

- **Point Anomalies**: occur when a single individual entity has observed irregular behavior from the rest of the data [37].
- **Contextual Anomaly**: An instance that could be considered anomalous in some specific circumstances is called a contextual anomaly, which is also called a conditional anomaly [36]. When a data value has irregular behavior compared to the rest of the data in a specific context, but not in all circumstances [38], therefore, if something is anomalous in some specific context, then it can be classified as a contextual anomaly.
- **Collective Anomalies**: often represent a collection of related entities as a correlated group that has observed anomalies against the remaining data. They are called collective anomalies [39].

## 8. Surveillance System

The surveillance system is a real-time administration program developed to identify and detect irregular activities directly automatically; it can be called an anomaly detection system [3]. Using advanced technology to manage crowds could be the proper approach to avoid any potential issues [40]. CCTV cameras are the most common safeguard instrument used to observe individuals and their activities. It is a typical policy to ensure safety. Approximately 770 million CCTV cameras have been installed around the world [41]. The constant observation of these cameras by humans is very difficult in a large crowd. The limitations of CCTV raised the requirements of continuous manual monitoring of the screens by the workers, which is very difficult to respond immediately to any actions and time-consuming. To overcome this limitation, an automatic system that could detect and identify abnormal behavior automatically and notify the authority to act at the same time is required. To detect the actions and categorize them effectively, deep learning techniques such as CNN, RNN, LSTM, and more are used, which produce outstanding results. This intelligent system is qualified to detect objects that differ significantly from the normal state, such as fighting, vagrancy, stampede, and incidents [42]. It is equipped in various areas, such as academies, roads, playgrounds, and hospitals, to encourage the management process [18].

## 9. Previous Reviews on Anomaly Detection

Several surveys have been published on crowd analysis and abnormalities detection. Some of the studies focus on general ideas and concepts, and some other research focuses on a specific area. As summarized in Table 1, this section presents some of the most significant reviews published between 2011 and 2022, which represent significant results and contributions. The main contribution of this review is the depth of concentration on the deep learning methods, role, and performance in human abnormalities detection in crowded areas. For dense crowds, a new taxonomic organization presents recent developments in human abnormal behavior detection. Furthermore, participate in the discovery of subfields that are still unexplored or that are rarely covered. Future directions and trends are demonstrated as open challenges for future research. This review focuses on studying the human crowd, specifically abnormal human behavior. 

## 10. Taxonomy of Anomaly Detection in Crowd Scenes

**Figure 4:**
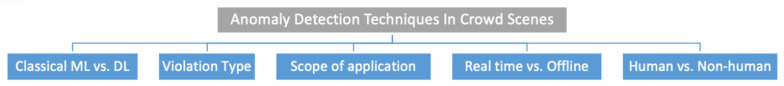
Anomaly detection techniques in crowd scenes.

With the increasing demand for security and safety of people in large-scale crowd areas, CCTV is used to monitor the crowds. Analyzing the video streams provided by CCTV is an important task to detect and localize the anomaly behavior in the crowd. However, the literature includes many studies that need to be classified to understand deep learning for efficient crowd management better. This section presents a classification of the previous studies according to classical ML vs. DL, anomaly type, the scope of application, real-time vs. offline, and human crowd vs. non-human crowd, as described in Figure 4.

### 10.1. Classical ML vs. DL

Classical ML is a collection of algorithms and techniques used to build a model that can learn from existing observations and exploit the learned data to predict new observations by finding patterns in data; it works better with small data [74,75,76,77,78,79,80,81,82]. DL is a subset of ML, which is based mostly on artificial neural networks [83,84,85,86,87,88,89,90,91,92,93]. When classical ML techniques are compared with DL techniques, the DL techniques achieve more significant performance and accuracy in many domains such as natural language, object detection, speech, image classification, and semantic segmentation [94]. ML acts satisfactorily on small to medium datasets, while DL acts sufficiently on large datasets. According to hardware requirements, ML can work on a local CPU, while DL requires powerful computing power such as a GPU. For feature engineering, ML needs to be explicitly identified or annotated features by humans, while DL can learn and discover features automatically by neural networks. For the training time, ML models usually take a short training time, while DL requires computationally intensive time and power for training. ML utilizes many automated algorithms that allow the model to generate predictions from employed data. While DL uses a quite different and complicated architecture called a neural network, that hands data via processing layers to interpret data and generate predictions. Current works on crowd anomaly detection can generally be classified into unsupervised, supervised, semi-supervised, and reinforcement learning. The dataset with both data and labels can be used for supervised anomaly detection. In anomaly detection, the labels indicate the type of event, whether it is “normal” or “abnormal”, or determine a specific type of anomaly such as fighting, burglary, and more. Unlabeled datasets have been employed for the unsupervised anomaly detection method. The unsupervised method considers that most of the events in the dataset are normal and otherwise assumed an anomaly. Semi-supervised anomaly detection techniques are used in situations where the dataset has not been completely labeled nor unlabeled, meaning that some data are labeled and some are unlabeled. The Supervision type column determines the type of the method: unsupervised [74,75,76,77,85,86], supervised [78,79,80,81,83,84,87,88,89,90,91,92], semi-supervised [82], and reinforcement learning [93]. The model column determines the algorithm used in work, CNN, RNN, GAN, KNN, SVM, GMM, …, etc. The anomaly column determines the type of anomaly that each study tries to detect. The dataset column names the dataset used in each study.

### 10.2. Violation Type

It is well known that the physical world produces abnormal behaviors that appear beyond explanation. Detecting these abnormal behaviors is not easy because it comes in several types. The studies [74,76,77,78] focus on detecting non-pedestrians and escape panics as abnormal behavior in a crowded place. Moreover, the study [79] includes more abnormal behavior such as irregular pedestrian movement and action differences from regular recognized events. It uses a single shot multi-box detector (SSD) to detect abnormal behavior in three different datasets PASCAL, VOC, and High-Speed Railway. The proposed improved SSD network achieved increased results on the three datasets by 2.52% and 4.74%, respectively. While [75] proposes a novel Gaussian kernel-based integration model (GKIM) for anomalous entities detection and localization in pedestrian flows. Then, a block-based detection framework was developed by training a recurrent conditional random field (R-CRF) using the GKIM features. This [75] study divides the detection process into two types, groups and individuals.

The group anomaly behavior includes suddenly scattered crowds, and individuals include non-pedestrians, escape panics, and action differences from regular recognized events. The proposed framework outperforms the compared methods in terms of equal error rate (EER) and detection rate (DR) in both frame-level and pixel-level with three different datasets UCSD, UMN, and UCD. A deep learning model that can detect normal or abnormal actions on an academic campus using CCTV footage has been introduced in [80], which uses three different datasets, UCSD, UMN, and LV, to detect the anomaly. The model consists of two parts and two neural networks, CNN and RNN: First, CNN is used for high-level feature extraction from video frames. Second, based on the obtained features, the RNN classifier predicts the class as normal or abnormal using LSTM architecture. A pre-trained model VGG-16 was used in image feature extraction with videos obtained from CCTV cameras. The results show that the introduced model allows for the prevention of crimes before it occurs. The real-time CCTV images were tracked and analyzed automatically and achieved an accuracy of 87.15%. Fighting and violence are the most common abnormal behaviors that occurred in public places, which are addressed for detection in [81,82,85,86,87,88,89]. The vehicles and bicycles that drive oppositely, at fast speed, or at not allowed places, for example, on the pedestrian side, which cues as dangerous for people, is considered abnormal behavior in [82,85,91,92]. In universities, specifically inside the campus, fighting, and fainting are irregular behaviors. An abnormal behavior recognition system based on 3D-CNN and LSTM has been developed in [87] to detect abnormal behavior in universities. The 3D-CNN and LSTM models are employed to maintain motion correlation between consecutive feature images using 3D-ResNets architecture. Crossing the track at the train station or railway outside the pedestrian zone is considered a wrong behavior that should be avoided [83,84].

### 10.3. Scope of Application

**Figure 5:**
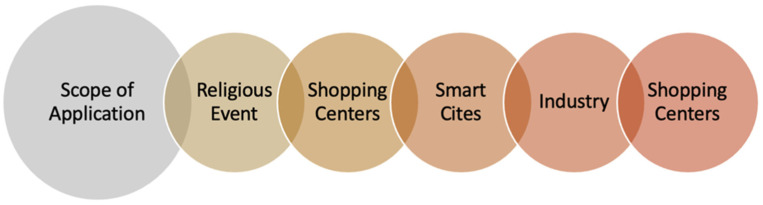
Scope of application.

Managing a large-scale crowd in crowded places is a business solution that offers an intelligent analysis of crowd mobility. It can be applied to applications that contain crowds, such as at shopping centers, queue detection, cultural events, public places theft detection, playgrounds, streets and highways, sports stadiums, train stations, and airport terminals, see Figure 5. For the Hajj aspect, an abnormal behavior detection approach based on optical flow and generative adversarial network (GAN) for crowd scenes anomaly detection has been proposed [27]. The optical flows are used to identify dynamic features. Then, an optical flow framework based on GAN has been employed with a transfer learning strategy to identify abnormal human behavior in large-scale crowd scenes during the Hajj. To differentiate between normal and abnormal behaviors, the U-Net and Flownet have been used. The suggested approach is evaluated using three datasets: UMN scenes 1, 2, 3, UCSD, and Abnormal Behaviors HAJJ datasets. The results indicate that the accuracy achieved with UMN scenes is 99.4%, 97.1%, and 97.6%, respectively.

Moreover, it achieves 89.26% with UCSD and 79.63% with the proposed Abnormal Behaviors HAJJ dataset. The model can work perfectly, but the accuracy requires to be enhanced by training the model with more samples and annotating more details. Moreover, a new crowd density prediction model for Hajj and Umrah crowd video analytics system has been proposed [95] to enhance the protection and safety of pilgrims in Makkah. CNN analyzed the crowd by counting the number of people in a specific area. The suggested model exceeds the state-of-the-art methods with a considerable decrease of MAE, which results in 240.0 and improved by 177.5 degrees, and MSE, which results in 260.5 and improved by 280.1 degrees, with the HAJJ-Crowd dataset. Indeed, COVID-19 is also spread by crowds, which are classified as sensitive sources. For crowd management during the pandemic, a recommendation system has been developed that suggests the closest shopping centers or stores with the least estimated crowds near the user’s location [96], which helps to avoid crowding and scrambling in stores. The top-K approach and behavioral game theory have been used to predict the user’s choice and estimate the crowd level for the requested place. The model outcomes indicate an increase in the trust factor from 0.5 to 0.76 and reduce the crowd level by an average of 40%. A lightweight CNN framework [84] has been proposed for anomaly detection in smart cities that is functional for a real-world surveillance environment. The introduced framework contains three key phases: First, the lightweight CNN model is used to extract spatial features from sequence surveillance video frames. Second, create a feature vector from a series of 30 frames of the video. Third, the feature vector is fed to the residual LSTM to identify abnormal activities in a real-world environment. The system’s outcome shows that using CNN features with the residual blocks in LSTM for sequence learning is effective for anomaly detection and recognition. A deep learning model that can detect normal or abnormal actions on an academic campus using CCTV footage has been introduced [80]. In case of an abnormal event, the model sends an alert message to the authority. The proposed model achieved an accuracy of 87.15% in abnormality detection in the academic campus area. Moreover, an industrial aspect required an intelligent real-time video surveillance system for anomaly detection to protect safety, which was developed in [97] and achieved good results. 

### 10.4. Real-Time vs. Offline

Locating moving objects in videos over time is known as object tracking [98]. It has a variety of applications in computer vision, such as analyzing human behavior in crowds [99,100], pedestrian tracking systems [101], body motion tracking in crowds [102], detecting anomalies in crowds [103,104], and monitoring traffic flow [105]. The capability to comprehend and model an object’s motion is crucial to the success of a tracker. Tracking can be performed with one object or several objects simultaneously. Even if an environment contains several objects, only a single object is tracked in single tracking regardless of how many objects are presented. While multiple object tracking involves observing all the objects in the environment over time [106]. However, tracking and abnormality detection are imperative, whether performed online in real-time or offline. An online approach gathers real-time data about people and their behavior to achieve an understanding of their behavior so that abnormalities can be detected immediately. Since the frames are processed at the same time of occurrence, the subsequent frames cannot be used to predict and improve results; only previous frames can. In another situation, offline trackers will be used when tracking an object in a stream that has been recorded. Using the previous and subsequent frames, the program conducts batch processing of the frames to analyze the video stream and provide accurate results. An intelligence system that controls the crowd by detecting abnormal behavior using deep learning techniques through a real-time video surveillance system has been introduced [107]. This system allows avoiding injury or any other action which causes harmful effects to the community because of the crowd using both CNN and KNN. A real-time lightweight computational architecture for violence detection in a crowded public place using convolutional long short-term memory (Conv-LSTM) has been developed [108]. A dataset of crowd anomalies was used to validate the algorithm, which achieved 95.16% accuracy.

Moreover, an efficient system that can detect and locate abnormal behavior in surveillance videos in crowded events has been introduced [109]. The proposed system is based on a new Motion Information Image (MII) model expressed using optical flow and CNN. The outcomes reveal that the introduced system is very efficient and can identify and locate abnormal behaviors in real-time. The algorithm’s accuracy outperforms the existing algorithms at both pixel and frame levels. In addition, a new system for real-time anomalous event detection in videos called MOVAD has been proposed [25]. It achieved comprehensive performance that exceeds the current state-of-the-art methods. Intelligent anomaly detection and classification systems were introduced in [85] to detect abnormal behavior in surveillance videos using Faster RCNN with Deep Reinforcement Learning (DRL) techniques for offline tracking. The proposed model has outperformed the other methods with the maximum accuracy of 98.50% and 94.80% on the test004 and test007 datasets. Another accurate and effective deep learning framework for detecting abnormal behavior in videos with Vgg-16 and LSTM has been developed [110]. Experimental results show that the proposed method achieves the best detection results at the frame and pixel levels. Indeed, a recurrent neural network (RNNs) and two-dimensional convolutional neural networks (2D CNN) have been developed for violence detection [111], that achieved an accuracy of 99%, 93.75%, and 96.74%, respectively, on the Hockey dataset, Violent Flow, and Real-Life Violence Situations Dataset.

Moreover, an abnormal behavior recognition system based on 3D-CNN and LSTM has been developed [87]. The 3D-CNN and LSTM models are employed to maintain motion correlation between consecutive feature images using 3D-ResNets architecture. The experiments show that the proposed method has an excellent performance in abnormal behavior recognition on some challenging datasets. An automatic abnormal behavior detection system of videos based on VGGNet and BSVM has been developed [112], it was applied through transfer learning strategies to detect abnormal events. The results illustrated that the VGGNet-19 network obtained better accuracy than other hand-crafted descriptors, with an average accuracy of 97:44%. A new fully convolutional neural networks (FCNs) architecture system for global abnormal behavior detection and localization in videos has been developed [113]. The proposed architecture is fast and accurate for anomaly detection in video data, which achieved a 370-fps processing speed on a standard GPU.

### 10.5. Human Crowd vs. Non-Human Crowd

The concept of “crowds” is not limited to specific objects. The term “crowds” can include different types of objects such as human crowds, vehicle crowds, crowds of animals, crowds of birds, and many more examples. A fundamental requirement for analyzing crowd scenarios is identifying the kind of crowd. Table 2 presents a summary of deep learning anomaly detection projects with different targets, including humans and non-humans. An anomaly detection system combining the optical flow method and convolutional neural network (CNN) has been introduced to identify and inform the irregularities of human and vehicle crowds in difficult video scenes [83]. The proposed system achieved an average accuracy of 86.3% and an average time of 12 s with the human crowd, while vehicle anomaly detection achieved 89.7% accuracy with an average time of 11 s. A novel architecture called DeepCrowd, which can detect and classify five different types of a crowd (vehicle crowd, human crowd, bird crowd, animal crowd, mixed crowd), has been developed [114]. The DeepCrowd system achieved a good accuracy of 83.11% in detecting and classifying the type of crowd. A unified autonomous system has been developed to detect risky human behavior in video surveillance systems or monitor systems RGB image based on a deep convolutional network [82]. The result shows the potential and possibility of the proposed system, which provides adequate achievement in distinguishing abnormal behavior in a real-world situation. For sparse crowds, an adaptive training-less method for anomaly detection in surveillance videos has been introduced [115]. It achieves comparable performance results with several state-of-the-art methods on publicly available UCSD, UMN, CUHK Avenue, and ShanghaiTech datasets.

## 11. Publicly Available Datasets for Crowd Applications

Large-scale applications for crowd management have received significant attention over the last ten years. For the management and control of crowds, crowd analysis is crucial in intelligent video surveillance systems. The collection of crowd motion video data is not an easy task. During the past few years, more and more datasets have been created that focus on crowd density estimation, crowd analysis, and anomaly detection in crowded scenes. The use of these datasets allows for improving the quality of crowd applications. Table 3 presents a summary of publicly available crowd datasets.

## 12. Discussion

A review examining recent research in crowd anomaly detection in automated surveillance systems has been presented in this paper, which includes the key aspects of the problem domain, approach, and method. Since video surveillance systems are widely used in public places, crowd anomaly detection has become an increasingly critical part of the intelligent video surveillance system. In intelligent video surveillance, anomaly detection and localization remain challenging problems. The definition of the anomaly is significantly different from one situation to another, which means anomalies in a specific situation may not be an anomaly in another situation. Consequently, the type of event depends on the surrounding circumstances. Several types of abnormal behaviors depend on the environment and circumstances, making detecting them difficult. To discover these behaviors, it is necessary to understand the surrounding environment and the expected and unexpected behavior to facilitate the classification of any other behavior that may occur. The anomaly behavior varies according to the environment; specifying the behavior more precisely ensures a more accurate discovery of the events. According to Table 2, most of the studies in this area focus on one target, abnormal behavior detection, and few studies investigate multiple targets. However, addressing multiple targets is very common as a realistic scenario and can be challenging since each target must be addressed with a different feature extraction method.

Furthermore, most human anomaly detection applications cover a range of viewing fields from around 10 M to a medium size area of around 100 M. It is very rare to find some applications that examine the effects of human abnormalities detection in very small or very large fields of view, such as that seen in a satellite image. In addition, object occlusions, inconsistencies in perspective due to different angles and camera positions, as well as tilting and up-and-down motion of the camera, can occur in large-scale crowd scenes, making crowd analysis very challenging. The use of multiple CCTVs that cover complete angles to monitor the crowd and provide a full (360°) view of objects in that area to avoid occlusion. Furthermore, drones and satellite images will add more valuable results during crowd monitoring and abnormality detection. According to the literature and previous applications, classical machine learning techniques are often outperformed by deep learning techniques. Machine learning algorithms can still provide good results by training the model well, but deep learning algorithms provide more accurate results in large and complex applications for greater accuracy. However, simple, direct, and clear requirements projects can be performed with machine learning algorithms without wasting resources. Indeed, some of the literature on video surveillance systems does not provide an exact distinction between real-time anomalous event detection and offline anomalous detection. The two approaches are different from each other in the data processing. Offline video tracking involves analyzing sequential video frames and relating target objects based on their appearance in each frame. While real-time object tracking involves tracking moving objects in video from a camera over time. Moreover, many types of objects can be characterized as crowds. The term crowd does not refer to any single type of object. Identifying the nature of the crowd is a prerequisite for analyzing crowd scenarios. It could be a crowd of people, vehicles, animals, or any other object.

## 13. Trends and Future Works

Detecting abnormal crowd behavior in video scenes is a hot research topic currently. Additional developments and improvements will help to achieve effective crowd management and ensure human safety. Video anomaly detection has a wide range of potential application domains such as crime detection, pedestrian tracking systems, traffic violations, body motion tracking in crowds, abnormal crowd behavior, intrusion detection, fraud detection, abandoned objects, health monitoring, weapons at sensitive areas, and industrial protection. For future research, the ability to analyze crowd behavior at the macro and micro levels will generate valuable information to understand and manage crowd behavior. The research on large-scale crowd object detection still needs further development. In a dense crowd, it is not easy for the object detector to pinpoint the position where events happen in video frames. Additional improvement in this aspect will produce an accurate detection result. Most of the research has recently focused on supervised approaches while monitoring the real environment produces large streams of unannotated data. Therefore, there is a need to improve the unsupervised real-time anomaly detection applications. New research areas have been identified, especially related to the crowds during pandemics the world is suffering.

Moreover, the use of adaptive deep networks is an advanced solution that utilizes real-time data to detect the anomaly. Exploring and determining the specific type of abnormal behavior for multiple people in the crowd under more diverse situations is another open challenge. Another important aspect of most deep learning projects, specifically for anomaly detection in crowds, is how to optimize and improve the model’s performance during running time. This point opens another chance for achieving a competitive detection time in the crowd. Further improvements are required for low resolution, illumination variation, and occlusion in data. Finally, the researchers emphasize the importance of advancing the topic quickly and appropriately. 

## 14. Conclusions

Over the last decade, CCTV surveillance has become more prevalent in crowded public places. This has led to more video data being produced than can be analyzed by an individual. Therefore, automated systems are necessary for analyzing large volumes of video streams in crowded areas to detect anomalies, ensure safety, and respond quickly. This paper reviews the recent development of automated anomaly detection systems from the perspective of computer vision. In addition, the taxonomic organization of existing works in crowd analysis and anomaly detection has been introduced. Previous applications provided great applications in detecting abnormal behavior. However, there is still a greater necessity to obtain higher performance and greater accuracy in detecting anomalies in crowded areas. Even though there have been numerous studies on detecting abnormal human behavior, more research is still needed to address numerous issues. Crowd abnormal behavior detection should be more accurate and robust against different situations in large-scale and heterogeneous crowds. Using advanced technology in monitoring the crowd, such as drones and satellites, will provide additional valuable insight.

## Figures and Tables

**Table 1:** Summarized presentation of review papers in anomaly detection.

Ref.	Year	Focus
[43]	2011	Computer vision techniques for analysis of urban traffic
[44]	2012	Anomaly detection in automated surveillance systems
[45]	2012	Detecting abnormal human behavior in the context of a video
[46]	2012	Discuss frameworks for recognizing human activity
[47]	2012	Human behavior analysis with semantic enhancement
[48]	2013	Intelligence video surveillance system (IVSS) using a multi-camera network
[49]	2014	Machine learning techniques for novelty detection
[50]	2015	Describe the difficulties that come with modeling for video anomaly detection
[51]	2016	Currently available anomaly detection video datasets issues
[52]	2017	Computer vision techniques used for crowd disaster avoidance
[53]	2017	Computer vision techniques for analyzing dense crowd scenes
[54]	2017	Explore various available methods used to identify abnormal crowd behavior
[55]	2017	Crowd statistics and behavior understanding
[56]	2018	Implementation of deep learning techniques for video anomalous detection
[57]	2018	Available methods for human abnormal behavior detection
[58]	2018	Unsupervised- and semi-supervised learning-based for video anomaly detection
[59]	2018	Feature extraction and description techniques for abnormal behavior recognition
[17]	2019	Deep-learning-based anomaly detection techniques for various domains
[60]	2019	Object trajectories, clustering, anomaly detection, summarization, and synopsis generation
[61]	2020	Video anomaly detection in road traffic
[62]	2020	Deep learning-based methods for analyzing crowded scenes
[63]	2021	Deep learning technique used for anomaly detection
[64]	2021	State-of-the-art deep learning-based approaches for detecting video abnormalities
[2]	2021	Explore various studies related to crowd analysis
[42]	2021	Deep learning-based algorithms for recognizing video anomalies, opportunities, and challenges
[65]	2021	For security systems, automated and real-time surveillance technologies of irregular action recognition are used to identify dynamic crowd behavior
[66]	2021	Analyzed and compared crowd anomaly detection methodologies
[67]	2022	Crowd count, human detection and behavior, anomaly detection, and importance of crowd analysis
[68]	2022	Crowd modeling and analysis
[69]	2022	Comparative analysis of existing crowd behavior analysis methods
[70]	2022	Deep learning framework for anomaly detection
[71]	2022	GAN-based anomaly detection
[72]	2022	Summarization of video analytics deep learning techniques in the Hajj scenes
[73]	2022	Evolution of anomaly detection methodologies in intelligent video surveillance

**Table 2:** Categorization of the state-of-the-art anomaly detection methods in crowd scenes.

Ref.	Type	Approach	Anomaly	Scope	Processing	Target	Dataset
**Classical Machine Learning**							
[74]	Unsupervised	K-means	Non-pedestrians, escape panics	Public Places	Offline	Human	UCSD, UMN
[93]	Unsupervised	Dictionary learning	Suddenly scattered, non-pedestrians, escape panics	Public Places	Offline	Human	UCSD, UMN PETS2009, Avenue
[92]	Unsupervised	Soft Clustering	Non-pedestrian, escape panics	Public Places	Offline	Human	UMN, UCSD
[91]	Unsupervised	k-means	Non-pedestrian	Public Places	Offline	Human	UCSD
[89]	Supervised	Optical flow	Non-pedestrians, escape panics	Public Places	Offline	Human	UCSD, UMN
[75]	Supervised	GKIM, R-CRF	Non-pedestrians, panics, irregular movement	Public Places	Offline	Human	UCSD, UMN, UCD
[76]	Supervised	K-means, Linear SVM	Crowd running, crash, kidnap, burglary, fighting	Public Places	Offline	Human	UCSD, UMN, LV
[77]	Supervised	SVM	Panics, fighting, running, standing	Public Places	Offline	Human	UMN, BEHAVE
[78]	Semi-Supervised	GMM, SVM	Violent, panics	Public Places	Real-Time	Human	UMN, Violent flows
**Deep Learning**							
[79]	Supervised	SSD, VGG-16	Bullet train, pedestrian	Railway	Offline	Human Train	PASCAL VOC, Railway
[90]	Supervised	SSD, VGG-16	Small object	Railway	Real-time	-	ILSVRC CLS-LOC, Railway
[88]	Unsupervised	GAN	Biking, fighting, vehicle, running	Public Places	Offline	Human Vehicle	CUHK Avenue UCSD, Campus ShanghaiTech
[87]	Unsupervised	3D-CNN LSTM	Panics, fighting, protest	Public Places	Offline	Human	UMN, CAVIA, Web
[94]	Supervised	Modified 3D ConvNet	Violent	Public Places	Offline	Human	Crowd violence
[80]	Supervised	CNN RNN	Use mobile in class, fighting, fainting	University	Offline	Human	KTH, CAVIAR
[81]	Supervised	CNN	Walking, jogging, fighting, kicking, punching	Public Places	Offline	Human	CMU, UTI PEL, HOF WED
[82]	Supervised	VGG-16 LSTM	Kicking, pointing punching, pushing	Public Places	Offline	Human	UT-Interaction-Data
[83]	Supervised	Optical Flow CNN	Panic, running fast speed, crash	Public Places	Offline	Human Vehicle	UCSD, UMN
[84]	Supervised	CNN Residual LSTM	Fighting, explosion, accidents, shooting, robbery, shoplifting, burglary	Smart Cities	Real-Time	Human	UCF-Crime, UMN, Avenue
[85]	Reinforcement Learning	Faster RCNN	Car, bicycle	Surveillance System	Offline	Vehicle	UCSD
[25]	Supervised	CNN, RNN KNN, Optical Flow	Bicycles, skateboards, wheelchairs	Public Places	Real-Time	Human vehicles	CUHK Avenue UCSD, campus, ShanghaiTeh, UR fall
[27]	Supervised	Optical Flow GAN	Standing, sitting, sleeping, running, moving in opposite, non-pedestrian	Hajj	Real-Time	Human Cars Wheelchairs	UMN, UCSD, HAJJ datasets
[95]	Supervised	CNN	Density	Hajj, Umrah	Real-Time	Human	HAJJ-Crowd
[96]	-	point-of-interests (POI)	Crowding, scrambling	Shopping Centers	Real-Time	Human	-
[97]	Unsupervised	CNN, Conv-LSTM	People littering, skateboard, Discarding items, loitering	Industrial	Real-Time	Human	CUHK Avenue UCSD Ped 1 UCSD Ped 2
[107]	Supervised	CNN, KNN	Injury	Public Places	Real-Time	Human	UMN
[108]	Supervised	Conv-LSTM	Violence	Public Places	Real-Time	Human	Standard crowd anomaly
[109]	Supervised	CNN, MII Optical Flow	Escape or panic situation	Public Places	Real-Time	Human	UMN PETS2009
[110]	Unsupervised	Vgg-16 and LSTM	Non-pedestrian	Public Places	Offline	Human Cars	UCSD Ped2 CUHK Avenue
[111]	Unsupervised	RNN, 2D CNN	Violence	Public Places	Offline	Human	Hockey, Violent-Flow, Real-Life Violence Situations
[112]	Supervised	VGGNet-19 BSVM	Running, Carts Bikers, Skateboarder	Public Places	Offline	Human	UMN, CSD-PED1
[113]	Supervised	FCNs	Car Skateboarder Wheelchair Bicycle, Wrong direction	Public Places	Offline	Human	UCSD, Subway
[114]	Supervised	2D CNN	-	Public Places	Offline	Vehicle, Human Animal, Bird Mixed	CVML Crowd Variety
[115]	Supervised	Optical Flow	Panics, loitering, running, throwing objects	Surveillance System	Offline	Human	UCSD, UMN CUHK Avenue ShanghaiTech

**Table 3:** Summary of available crowd datasets.

Ref.	Year	Name	Scale	Train	Test	Total	Description
[116]	2021	CVCS	Medium	-	-	31	Multi-view crowd counting
[117]	2021	DroneCrowd	Large	-	-	112	Detection, tracking, and counting animal crowds with drones
[27]	2020	HAJJv1	Large				Human abnormal behavior in Hajj
[118]	2020	UCF-QNRF	Large	-	-	1535	Crowd counting and localization
[119]	2020	NWPU-Crowd	Large	-	-	5109	Crowd counting and localization
[120]	2019	DLR-ACD	Large	-	-	33	Crowd counting, density estimation, and localization
[121] [122]	2019 2020	JHU-CROWD JHU-CROWD++	Large	-	-	- 4372	Crowd counting dataset under different weather conditions
[123]	2018	CrowdFlow	Large	-	-	10	Crowd analysis, crowd flow, and movement estimation
[124]	2018	SCUT-HEAD	Large	-	-	4405	Head detection
[125]	2018	SmartCity	Large	-	-	50	Crowd counting
[126]	2017	Multi-Task Crowd	Large	-	-	100	Crowd counting, violence detection, and density level classification
[127]	2016	Shanghai Tech Part A Part B	Large	-	-	482 716	Crowd counting and density estimation
[128]	2015	WorldExpo ’10	Large	-	-	3980	Crowd counting in a cross-scene
[129]	2015	WWW Crowd	Large	-	-	10,000	Crowd understanding
[130]	2015	SHOCK	Large	-	-	-	Analyze spectator crowd behavior at stadiums/theaters/events
[131]	2014	CUHK Crowd	Large	-	-	474	Analyze group behavior in crowd scenes.
[132]	2014	Crowd Saliency	Large				Crowd movement, counter flow, source, sink, and instability motion
[133]	2013	UCF-CC-50	Large	-	-	50	Extremely dense crowd dataset for crowd counting
[134]	2012	AGORASET	Large	-	-	-	Crowd motion simulation
[135]	2012	Violent flows	Large	-	-	246	Classify and detect violent and non-violent behavior
[136]	2012	Mall	Medium	-	-	2000	Crowd counting
[137]	2012	Grand Central	Medium	-	-	-	Crowd train station dataset
[138]	2009	PETS2009	Medium	-	-	875	Crowd counting, density estimation, tracking, and event detection
[139]	2009	UMN	Small	-	-	11	Abnormal crowd behavior detection
[140]	2008	UCSD Peds 1 UCSD Peds 2	Small	6800 2550	7200 2010	40 12	Abnormal crowd behavior detection
